# Aligning Patient and Surgeon Aesthetic Priorities in Autologous Breast Reconstruction: A Cross-Sectional Survey Study

**DOI:** 10.1055/s-0045-1811167

**Published:** 2025-10-01

**Authors:** Belén Andresen-Lorca, Iván Heredia-Alcalde, Pedro Alvedro-Ruiz, María García-García, Ana Trapero, María D Pérez-del-Caz

**Affiliations:** 1 Department of Plastic Surgery and Burns, University and Polytechnic Hospital La Fe, Valencia, Spain; 2 Department of Plastic Surgery and Burns, San Carlos Clinic Hospital, Madrid, Spain

**Keywords:** breast reconstruction, autologous breast reconstruction, abdominally based breast reconstruction

## Abstract

**Background:**

Currently, there is no published evidence on the aesthetic and functional aspirations of women undergoing autologous breast reconstruction. Recognizing that not all surgical goals can always be achieved, we aimed to develop a priority scale based on patient preferences to guide intraoperative decision-making.

**Materials and Methods:**

We conducted a cross-sectional survey targeting patients, plastic surgery specialists, and trainees. Participants ranked 10 aesthetic and functional aspects of breast reconstruction (volume, shape, symmetry, sensitivity, texture, scarring on the breast and abdomen, umbilicus appearance, nipple–areola complex reconstruction, and similarity to the original breast). Responses were analyzed using nonparametric statistical methods, using SPSS (IBM SPSS Statistics, IBM Corp., New York, United States.).

**Results:**

A total of 109 responses were collected (56 patients, 26 specialists, 27 trainees). Symmetry emerged as the top priority across all groups. Patients assigned higher importance than surgeons to sensitivity (6.77 vs. 4.38–4.50), nipple–areola complex reconstruction (8.18 vs. 7.72–7.78), and similarity to the original breast (6.55 vs. 4.63–4.89). Experienced surgeons valued breast texture more highly than less-experienced surgeons (8.67 vs. 6.70).

**Conclusion:**

While symmetry is universally prioritized, patients place greater value on functional aspects like breast sensitivity. These insights highlight the importance of personalized preoperative counseling to align surgical planning with patient expectations.

## Introduction

Breast cancer is one of the most common malignancies in female population around the world, affecting women in every country at any age after puberty. 1 Its treatment many times involves complete excision of the mammary gland, resulting in mutilation of the breast mound.

For many years, these patients had little or no options for reconstruction. Sequelae from a mastectomy or lumpectomy with adjuvant radiotherapy were permanent and served as a constant reminder of their battle against breast cancer. 2 However, these days, breast reconstruction is considered as one more step in the global treatment of this malignancy. It has evolved from an initial seek of merely covering and restoring the chest wall to the actual possibility of reproducing a naturally looking breast with a nipple–areola complex (NAC), searching for symmetry to the contralateral healthy mammary gland. 3

In this sense, autologous reconstruction, particularly the one using abdominal tissue (deep inferior epigastric perforator flap [DIEP]), has become the *gold standard* in breast reconstructive surgery. As for any other kind of reconstruction, the plastic surgeon will aim to achieve the best possible results in the reconstructed region (breast) with the minimal donor site (abdomen) violation. This involves trying to attain optimal breast shape, volume, texture, and symmetry, caring at the same time about the aesthetics of the imprints we leave behind on the abdomen.

However, in some cases, patients' anatomical characteristics, conditioning factors derived from previous surgeries, or others limit our possibilities, forcing us to prioritize some of the above-mentioned aspects over others. In order to decide which of these aesthetic goals we should prioritize and which could be somehow neglected, it appears reasonable to investigate what women's aesthetic aspirations are when they decide to subject themselves to reconstructive breast surgery. Indeed, a revision of the published literature will bring us to numerous studies on patient-reported outcomes (PROMs) 2 4 following breast reconstruction; however, there is little or no evidence on their aesthetic preferences. With the intention to build a value scale that could guide our decisions when performing autologous breast reconstruction, we decided to move our focus from the moment “after” (PROMs) to the one “before” reconstruction, changing our question from “what do you think about the result of your surgery?” to “what do you want to achieve for your breast with this surgery?”.

## Materials and Methods

Using the platform *QuestionPro* , we built a survey that was telematically distributed to three groups of participants: patients, residents/fellows, and plastic surgery specialists. The survey was composed by a number of preliminary questions on demographic data (different for each group), followed by one main question evaluating 10 aspects of autologous breast reconstruction, which were asked to be rated from 1 to 10 according to their relevance for the interrogated subject:

- Breast volume.
- Breast shape.
- Symmetry to the contralateral breast.
- Sensitivity of the reconstructed breast.
- Breast texture.
- Scars on the reconstructed breast.
- Scars on donor site (abdomen).
- Aspect of the transposed umbilicus.
- Reconstruction of the NAC.
- Similarity to the original breast (prior to mastectomy).

In order for each item to be correctly understood, especially for the case of patients, they were each accompanied by a short explanation clarifying exactly what we were referring to, as well as a simple self-made drawing to help them visualize what was being asked. Also, “sensitivity” was explained to patients as “the ability to notice tactile stimuli on the reconstructed breast, rather than the presence of erogenous sensation.” This aimed to ensure a consistent understanding of the functional nature of sensitivity.

Although the questionnaire did not undergo formal psychometric validation, several strategies were implemented to ensure clarity and consistency — particularly for patient respondents. These included pilot testing with a small group of postoperative patients, iterative feedback from clinical colleagues, and the incorporation of explanatory text and schematic illustrations for each item, facilitating comprehension by patient respondents. ( Appendix 1 [available in the online version]).

Medical records from patients reconstructed by our unit during the last 10 years were reviewed. Patients were contacted by telephone and asked to participate by sharing their e-mail directions, to which an online link for the survey would be sent for them to fill out anonymously. Spanish and English versions of the survey were also distributed amongst national and international specialist surgeons and surgeons in training. Colleagues were contacted via social media or messaging platforms. They were informed about the nature of the study and asked to participate anonymously.

Answers were collected automatically through the survey's server ( *QuestionPro* ) and later exported to SPSS for statistical analysis. A Shapiro–Wilk test was first performed, showing the lack of normality of our sample. Consequently, nonparametric tests were applied. A Kruskal–Wallis test was used to compare means between the three groups. Mann–Whitney U-tests were employed to perform intra-group comparisons (elder vs. younger patients and more vs. less experienced surgeons).

## Results

Our response rate was 68% for patients, 74% for specialist surgeons, and 60% for surgeons in training, collecting in total 56 answers from patients, 26 from specialist surgeons (Spanish and international), and 27 from surgeons in training (residents or fellows, both Spanish and international). Patient, “specialist surgeon,” and “surgeon in training” sample characteristics are summarized in Supplementary Table S1 (available in the online version).

Our patient sample came entirely from Hospital La Fe in Valencia, Spain. Their average age was 52.67 years, with a median of 52, indicating a generally middle-aged population. They had all been already operated on at the time of response.

The group of specialist surgeons consisted of 26 individuals, 69.2% men and 30.8% women. The majority were Spanish (76.9%), while the rest came from various other countries (France, Czech Republic, United Kingdom, and United States). In terms of experience, 34.6% handled fewer than one case per month, 26.9% performed at least one case monthly, and others had a higher frequency, with 15.4% performing more than one case weekly. The total number of cases each surgeon had reconstructed varied widely, with 26.9% having completed fewer than 10 cases, while 11.5% (3 out of 26) had completed more than 300 cases.

Finally, for the group of surgeons in training, 27 responses were collected, consisting of nearly equal numbers of men (51.9%) and women (48.1%), with a majority of Spanish nationals (77.8%), while the remaining trainees came from various European countries (France, Belgium, Austria, Italy, and the United Kingdom). Experience levels varied, with the majority being third and fourth-year residents and only one fellow. Their surgical experience was diverse. Cumulatively, nearly half of the trainees had assisted in 20 to 50 cases, while smaller groups counted fewer than 10 or more than 50 cases assisted.

**Fig. 1:**
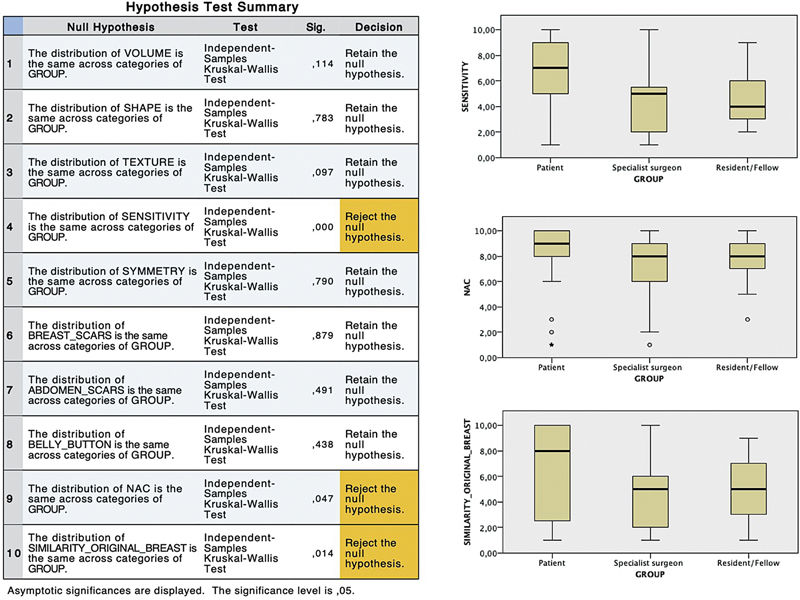
Comparison between patients, specialists, and surgeons in training.

There was congruence amongst all three groups for the highest-priority aspect of autologous abdominally based breast reconstruction: symmetry. However, differences emerged between patients and surgeons (both specialists and trainees) in relation to the item considered lowest priority: patients ranked the umbilicus as least important, whereas surgeons prioritized sensitivity lowest ( Tables 1 2 3 ). Also, we noted statistically significant differences in the ratings patients or surgeons gave to 3 of the 10 evaluated items ( Fig. 1 ): sensitivity, reconstruction of the NAC, and similarity to the original breast (prior to undergoing mastectomy).

**Table 1:** Patient results

Item	Mean evaluation (/10)
Volume	7.46
Shape	8.39
Texture	7.23
Sensitivity	6.77
Symmetry	9.14
Scars on recipient site (breast)	7.23
Scars on donor site (abdomen)	7.02
Belly-button	6.29
Nipple–areola complex (NAC)	8.18
Similarity to original breast (pre-mastectomy)	6.55

**Table 2:** Specialist surgeon results

Item	Mean evaluation (/10)
Volume	7.72
Shape	8.54
Texture	7.15
Sensitivity	4.50
Symmetry	9.35
Scars on recipient site (breast)	7.69
Scars on donor site (abdomen)	7.88
Belly-button	7.20
Nipple–areola complex (NAC)	7.12
Similarity to original breast (pre-mastectomy)	4.63

**Table 3:** Surgeon in training (resident/fellow) results

Item	Mean evaluation (/10)
Volume	6.78
Shape	8.93
Texture	6.33
Sensitivity	4.38
Symmetry	9.33
Scars on recipient site (breast)	7.59
Scars on donor site (abdomen)	7.26
Belly-button	6.93
Nipple–areola complex (NAC)	7.78
Similarity to original breast (pre-mastectomy)	4.89

For intra-group analysis, patients were divided into two groups placing the cut-off point on the sample's median age (52). Mean ratings for the 10 evaluated items were compared between both groups (over or under 52), finding no statistically significant differences between them.

**Fig. 2:**
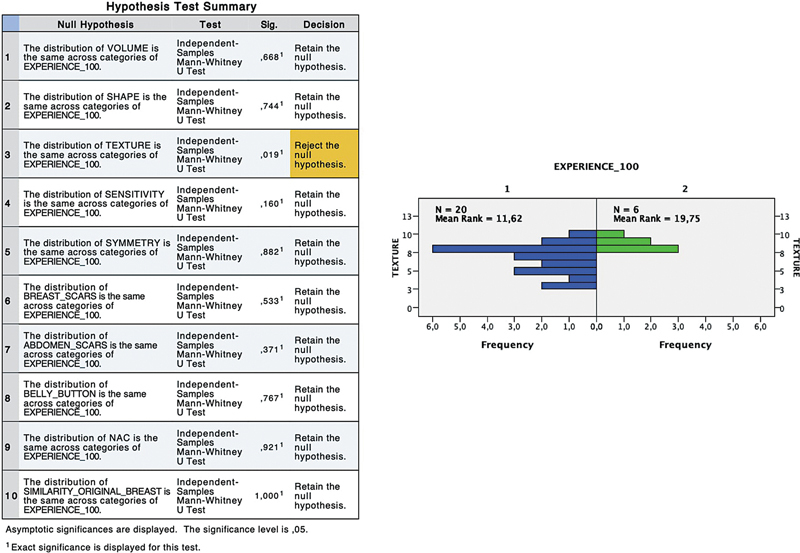
Intragroup comparison (specialist surgeon).

Analogous comparisons were made for the “specialist surgeon” sample, with an intention to evaluate eventual disagreements among them depending on their experience. In this case, the sample was divided into two groups based on the total amount of autologous reconstructions performed by each. Those having operated less than 100 cases throughout their careers were allocated to the first group (less experienced) and the ones having performed 100 or more to the second group (more experienced). Differences were observed only for “texture,” which more experienced surgeons valued with significantly higher marks (8.67 vs. 6.70) than less experienced ones ( Supplementary Table S2 [available in the online version]). Indeed, all surgeons having performed more than 100 breast reconstructions rated “texture” with more than an 8/10, whereas for the other group only 9/20 surgeons gave the mentioned item such importance ( Fig. 2 ).

## Discussion

Mastectomy exerts a profound impact on women both physically and emotionally, as it significantly affects body image, self-esteem, and overall quality of life. 5 6 This surgical procedure frequently presents psychological challenges, largely due to the loss of a body part intrinsically linked to femininity and identity. Given that breast cancer remains the most commonly diagnosed cancer among women worldwide, with millions of new cases each year, 1 the necessity for effective treatment and comprehensive support strategies, including reconstructive surgery options, cannot be overstated.

In this regard, breast reconstruction plays a pivotal role, as it not only restores the shape and volume of the breast but also contributes to the patient's self-image and psychological well-being. Among the available reconstructive techniques, autologous breast reconstruction using abdominal tissue—DIEP flap—is widely acknowledged as the *gold standard* . 3 7 8 9 10 11 This preference is supported by extensive clinical evidence demonstrating its superior outcomes over alternative methods. Furthermore, the increasing focus on PROMs 2 4 12 has provided a more nuanced and comprehensive perspective on the advantages and limitations associated with this technique. Despite the wealth of evidence, 13 14 15 a notable gap persists in understanding the specific aesthetic priorities that patients value most when undergoing this procedure.

The present study seeks to address this gap by investigating patients' aesthetic concerns and establishing a priority scale informed by their expectations and values. Such an approach is crucial, as achieving all aesthetic goals may not always be feasible due to anatomical or surgical limitations. Consequently, effective and personalized surgical planning is often required to balance competing priorities and optimize outcomes.

Our findings underscore the paramount importance of achieving breast symmetry in autologous reconstruction. This aspect was universally identified as the most critical parameter by patients, specialist surgeons, and trainees alike. This consensus is consistent with existing literature, 16 which highlights breast symmetry as a key determinant of aesthetic success and long-term patient satisfaction.

However, opinions diverged significantly regarding the least important parameter. While patients ranked the appearance of the umbilicus as the least significant, surgeons, including specialists and trainees, considered sensitivity to be of lesser priority. This discrepancy can be attributed to differing perspectives: surgeons' heightened concern for abdominal aesthetics, shaped by their experience with procedures such as lipoabdominoplasty, contrasts with patients' view of abdominal improvements as a secondary benefit rather than a primary objective.

Conversely, patients placed greater importance on breast sensitivity, reflecting their aspiration for reconstructed breasts to approximate natural characteristics, including protective and, ideally, erogenous sensation. However, surgeons, cognizant of the technical challenges and limitations in achieving such outcomes, often relegated this aspect to a lower priority. Notably, advancements in flap reinnervation techniques have shown promising potential for enhancing sensitivity outcomes. 17 18 19 20 21 This trend is reflected in our findings, which revealed that more experienced surgeons assigned higher importance to sensitivity compared to their less experienced counterparts, although with no statistical significance.

Moreover, statistically significant differences emerged between patients and surgeons regarding the importance of sensitivity, the reconstruction of the NAC, and the resemblance of the reconstructed breast to its pre-mastectomy appearance. Patients consistently rated these aspects higher than surgeons. The lower priority assigned to NAC reconstruction by specialists may be explained by their perception of this procedure as relatively straightforward and noncritical. However, for patients, the emotional significance of reconstructing the NAC is substantial. Similarly, the high value patients placed on recreating the original appearance of their breasts underscores the emotional resonance of this feature.

Interestingly, no age-related differences were observed among patients regarding their priorities. However, surgeon experience significantly influenced the perceived importance of breast texture. Experienced surgeons placed greater emphasis on achieving a supple and natural breast texture, reflecting their refined skills and heightened expectations. In contrast, less experienced surgeons appeared more forgiving of suboptimal textural outcomes, possibly due to the challenges of detecting such deficiencies in before-and-after photographs.

It is worth noting that patients uniformly assigned higher importance to all aspects of reconstruction compared to surgeons, with average scores ranging from 6.29 to 9.14. This finding suggests that patients perceive every aesthetic and functional feature as meaningfully contributing to their overall satisfaction.

Remarkably, patients exhibited less concern about donor site scars, a preference that could provide surgeons with greater flexibility in flap positioning. For example, this flexibility might allow for the placement of the lower abdominal scar slightly higher to facilitate a more reliable and manageable free flap and ensure safer donor site closure.

While our study focused on preoperative priorities, existing PROMs such as the BREAST-Q assess postoperative satisfaction. Several domains evaluated by BREAST-Q—such as symmetry, nipple reconstruction, and sensation—align with our findings. Thus, our results complement PROM-based research by offering insight into expectations that may shape satisfaction.

In conclusion, our findings indicate that surgeons are generally well aligned with patients' priorities, particularly regarding the importance of breast symmetry. However, surgeons could benefit from reevaluating the emphasis placed on other aspects, such as sensitivity, to better address factors that contribute to long-term satisfaction. These insights are invaluable for optimizing surgical procedures and managing patient expectations more effectively.

Nevertheless, it is important to acknowledge the limitations of this study. First, the patient population was derived from a single institution, which may limit the generalizability of the findings. Surveying patients from multiple centers was not feasible due to ethical and logistical constraints, including the need for local approvals and controlled patient access. However, the surgeon sample included both national and international participants—from countries such as France, Czech Republic, United Kingdom, and the United States—offering a broader view of training backgrounds and aesthetic values. Conversely, only 11% of the specialist sample could be classified as highly specialized in autologous breast reconstruction, having performed over 300 cases. This low proportion may skew the findings toward less experienced perspectives, which may not fully represent the practices and priorities of highly specialized surgeons. Finally, and most importantly, a major limitation is the potential selection bias, as only postoperative patients were surveyed, making their responses potentially influenced by their satisfaction or dissatisfaction with their outcomes, despite efforts to focus the survey on preoperative expectations. Future studies including preoperative assessments could help validate these findings.

## Conclusion

This study contributes to the field of autologous breast reconstruction by generating evidence on patients' aesthetic priorities when they decide to undergo autologous abdominally based breast reconstruction, aiming to support a more personalized and patient-centered approach. Understanding these preferences can help surgeons better align their surgical goals with patients' expectations, potentially improving satisfaction with aesthetic outcomes. While all groups agree on symmetry as the most important factor of the reconstruction, patients assign higher importance to a range of aspects, among which sensitivity stands out. This priority, which currently receives less focus from surgeons, suggests that a holistic approach to reconstruction is essential to meet patient expectations. The fact that more experienced surgeons do adopt strategies to sensitize the reconstructed breast proofs its significance. Our findings suggest that incorporating a structured preoperative counseling session, explicitly addressing functional outcomes such as sensitivity and aesthetic goals like symmetry and texture, may improve patient satisfaction and alignment of expectations.
