# Supplementary material for: Aligning Patient and Surgeon Aesthetic Priorities in Autologous Breast Reconstruction: A Cross-Sectional Survey Study
Source: Indian J Plast Surg. 2025 Oct 1;59(2):97–102. doi: 10.1055/s-0045-1811167 (PMC13290355; doi:10.1055/s-0045-1811167)
Supplement: Supplementary file 1 — Supplementary Appendix [file 10-1055-s-0045-1811167-s2513297a1.pdf]

Appendix 1 Survey instrument.

 © 2025. The Author(s).

ABDOMINALLY BASED AUTOLOGOUS BREAST RECONSTRUCTION (DIEP or msTRAM)

A survey directed to surgeons

Please indicate your sex:

- ◯ Man
- ◯ Woman
- ◯ NA

Please indicate the country in which you perform your surgical activity:

Please indicate the hospital you work in:

¿How many years have you been working as a surgeon since you finished your residency program?

- ◯ < 5 years
- ◯ 5 - 10 years
- ◯ 10-15 years
- ◯ 15 - 20 years
- ◯ > 20 years

How frequently do you perform breast reconstructions with abdominal free flaps (DIEP or msTRAM)?

- ◯ More than one case per week
- ◯ One case per week

- One case every 2 weeks
- At least one case every month
- Less than one case per month

More or less, how many breast reconstructions with abdominal free flaps can you calculate you have performed throughout your whole career?

- Less than 10
- 10 - 20
- 20 - 50
- 50 - 100
- 100 - 200
- 200 - 300
- > 300

Please rate the following items according to the importance you give to each of them during your abdominally-based free flap breast reconstructions (DIEP or msTRAM). You should rate each one of them with a mark from 1 to 10: **1 meaning IT IS NOT IMPORTANT TO YOU, 10 meaning IT IS VERY IMPORTANT TO YOU**. PLEASE READ ALL THE ITEMS BEFORE ANSWERING; IT WILL HELP YOU CHOOSE YOUR RESPONSE.

VOLUME

Is it important to you that the reconstructed breast has a given volume (either small or big, depending on the case)? Or do you believe that the final breast size is not that important? Choose 1 if you do not consider breast size to be important at all or 10 if it is very important to you that the breast size matches your patient's characteristics.

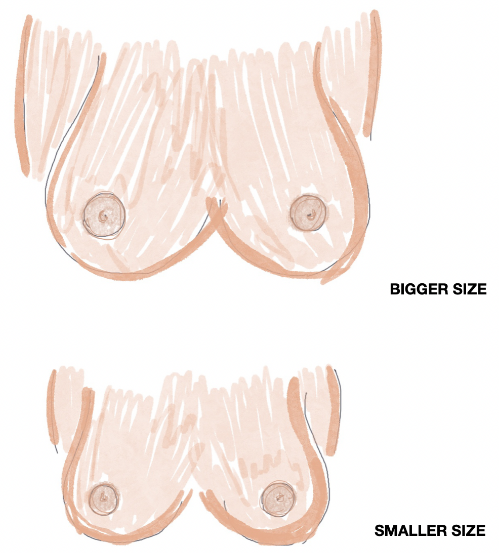

SHAPE

Is it important to you that the reconstructed breast has a given shape? Or do you believe its final shape (whatever it might be) doesn't matter that much? Choose 1 if you do not consider breast shape relevant or 10 if it is very important to you that the breast shape matches your patient's characteristics.

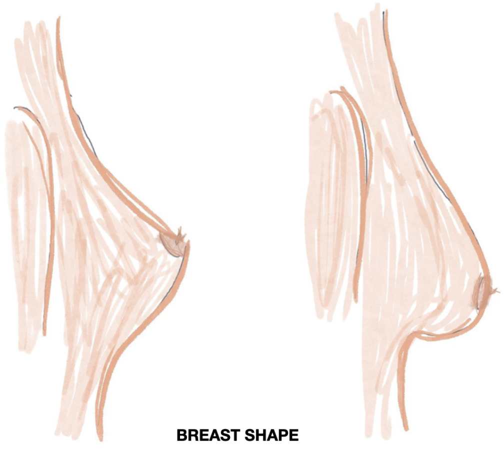

TEXTURE

Is it important to you that the reconstructed breast has a soft texture (as close as possible to an anatomical breast)? Or do you think it is not important whether it is soft or hard as long as it meets other criteria that might be more important to you? Choose 1 if you don't give any importance at all to the final breast texture or 10 if it is fundamental for you that it has de adequate texture.

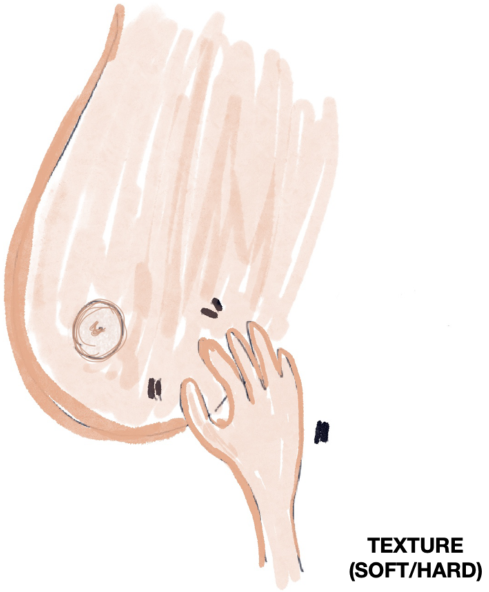

SENSATION

Is it important to you that the reconstructed breast has sensation (sensate reconstruction)? Choose 1 if you believe it is not necessary at all to perform a sensate free flap or 10 if it is essential for you that the reconstructed breast has the most sensation possible (even if this means increasing the complexity of your reconstruction).

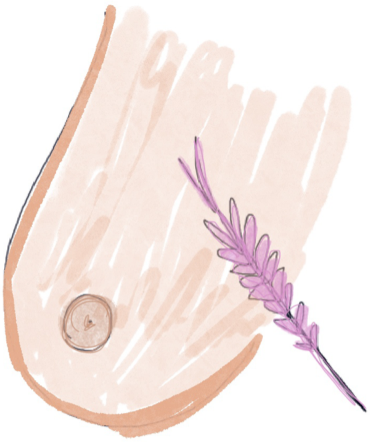

TOUCH (SENSATION)

QuestionPro

SIMMETRY

Is it important to you that the reconstructed breast is as similar as possible to the contralateral one? Or do you not worry about them being different? Choose 1 if you do not care at all about breast symmetry or 10 if it is a strong priority for you that the breast are as similar as possible (even if this means adding more surgeries - as many as necessary - to symmetrize them).

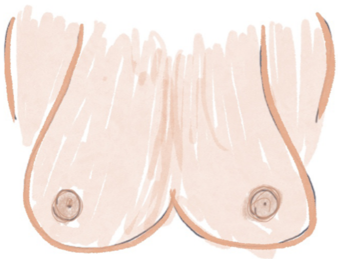

SYMMETRICAL

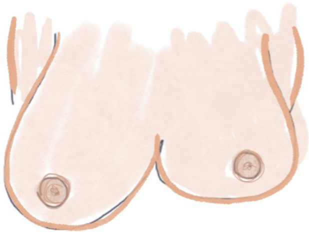

ASYMMETRICAL

BREAST SCARS

Is it important to you that the scars on the reconstructed breast are of good quality and as hidden as possible? Or do you not care about the scars as long as the breast meets other criteria that might be more important to you (shape, size, etc.)? Choose 1 if the scars on the breast have no importance to you or 10 if it is fundamental for you that they have the highest quality and are located as hidden as possible.

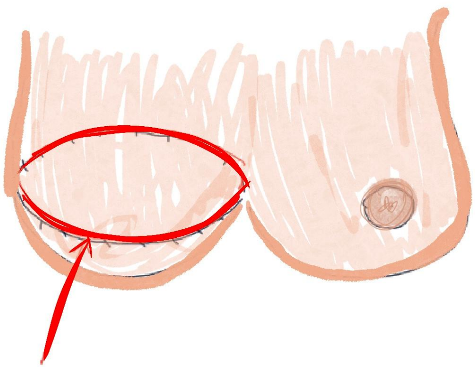

TUMMY SCARS

Is it important to you that the scars on the donor site (abdomen) are of good quality and as hidden as possible? Or do you not care about the scars as long as the breast reconstruction is the best possible? Choose 1 if the scars on the tummy have no importance to you or 10 if it is fundamental for you that they have the highest quality and are located as hidden as possible.

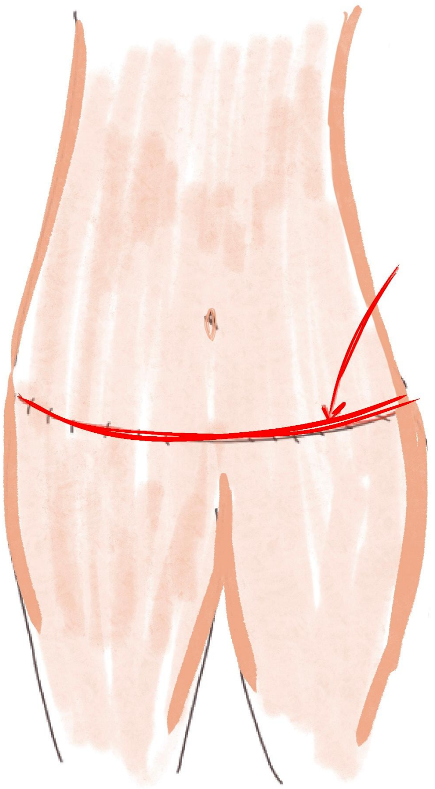

BELLY-BUTTON

Is it important to you for the patient's belly-button to be as aesthetic as possible after the reconstruction (considering its position, shape, size, etc.)? Or do you not care about how the belly-button looks as long as the reconstructed breast is the best possible?Choose 1 if you give no importance to how the belly-button looks or 10 if its final aspect is a big priority to you.

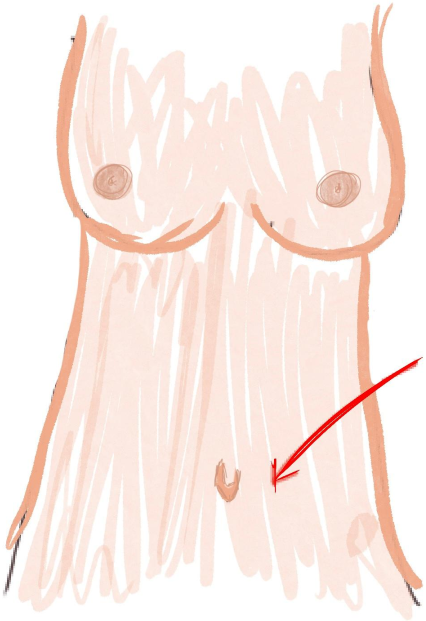

NIPPLE-AREOLA COMPLEX (NAC)

Is it important to you to include the reconstruction of the NAC in your breast reconstructions (regardless the moment this reconstruction takes place)? Or do you consider the NAC to be a minor/secondary aspect of the reconstruction? Choose 1 if you do not think reconstructing the NAC is important at all or 10 if it is fundamental for you to reconstruct it as well (regardless of the timing or the technique used).

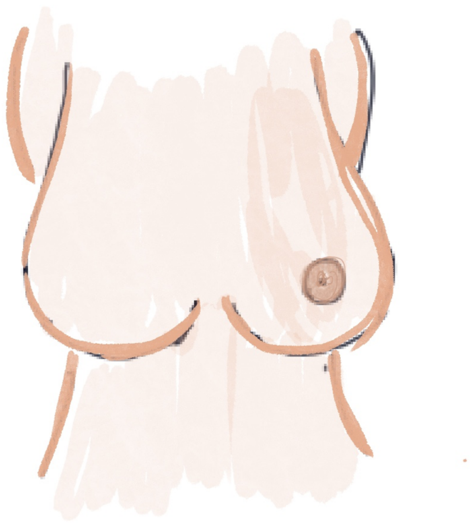

NIPPLE-AREOLA COMPLEX
NOT RECONSTRUCTED

RESEMBLANCE TO THE ORIGINAL BREAST (BEFORE MASTECTOMY)

Do you believe it is important that the final aspect of the reconstructed breast is as close as possible to the aspect of the breast before the mastectomy was performed (trying to simulate as much as possible its shape, size, fall, etc).? Or do you believe it is not important at all for it to be completely different to the original one as long as it meets other criteria that might be more important to you (shape, size, symmetry with the contralateral breast, etc).? Choose 1 if you give no importance at all to the fact that it is different to the previous one or 10 if it is a big priority for you to try and replicate the original breast.

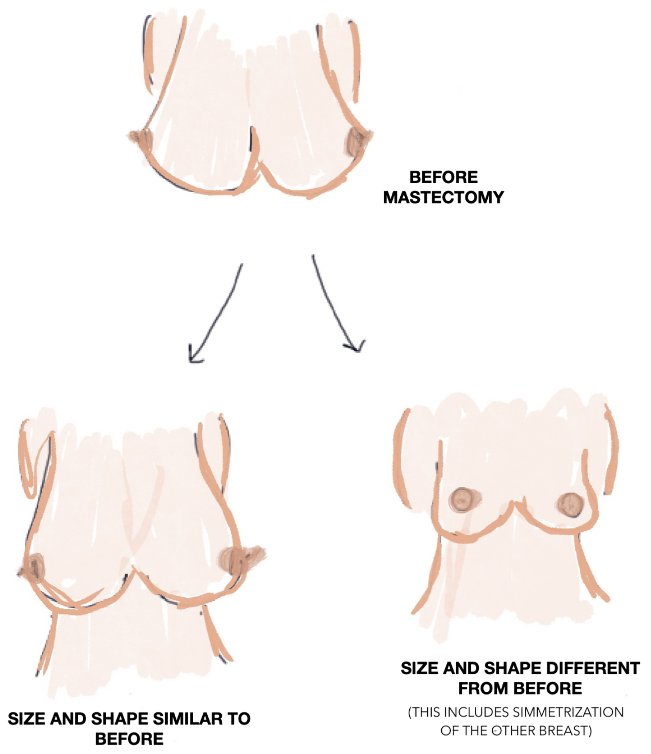

Taking into consideration the reconstructions you have performed during the last 1-2 years, how would you evaluate your overall results?

Very poorly | Poorly | Regular | Well | Excellent
