# Supplementary material for: Aligning Patient and Surgeon Aesthetic Priorities in Autologous Breast Reconstruction: A Cross-Sectional Survey Study
Source: Indian J Plast Surg. 2025 Oct 1;59(2):97–102. doi: 10.1055/s-0045-1811167 (PMC13290355; doi:10.1055/s-0045-1811167)
Supplement: Supplementary file 2 — Supplementary Table [file 10-1055-s-0045-1811167-s2513297.pdf]

Publisher: Thieme; Journal: IJPS; Article Type: Original Article
Journal ISSN: 0970-0358; Article ID Number: IJPS-25-1-3297
Volume Number: xx; Issue Number: xx

Supplementary Table S1 Patient, specialist surgeons, and surgeons in training sample characteristics

**Patients**	
N	56
**Age**	
**Mean**	52.67
**Median**	52
**Country**	Spain (56)
**Hospital**	Hospital La Fe, Valencia (56)
**Specialist surgeons**	
N	26
**Sex**	
**Men**	18 (69.2%)
**Women**	8 (30.8%)
**Nationality**	
**Spanish**	20 (76.9%)
**International**	6 (23.1%)
**Country**	
**Spain**	20 (76.9%)
**France**	3 (11.5%)
**Czech Republic**	1 (3.8%)
**United Kingdom**	1 (3.8%)
**United States**	1 (3.8%)
**Experience (years)**	
**<5 y**	11 (42.3%)
**5–10 y**	7 (26.9%)
**10–15 y**	5 (19.2%)
**15–20 y**	1 (3.8%)
**>20 y**	2 (7.7%)
**Frequency of interventions performed**	
**<1 case per month**	9 (34.6%)
**At least 1 case per month**	7 (26.9%)
**One case every 2 weeks**	5 (19.2%)
**One case every week**	1 (3.8%)
**>1 case a week**	4 (15.4%)
**Total # of cases reconstructed**	
**<10**	7 (26.9%)
**10–20**	7 (26.9%)
**20–50**	1 (3.8%)
**50–100**	5 (19.2%)
**100–200**	1 (3.8%)
**200–300**	2 (7.7%)
**>300**	3 (11.5%)
**Surgeons in training**	
N	27
**Sex**	
**Men**	14 (51.9%)

**Women**	13 (48.1%)
**Nationality**	
**Spanish**	21 (77.8%)
**International**	6 (22.2%)
**Country**	
**Spain**	21 (77.8%)
**France**	2 (7.4%)
**Belgium**	1 (3.7%)
**Austria**	1 (3.7%)
**Italy**	1 (3.7%)
**United Kingdom**	1 (3.7%)
**Experience (years)**	
**1st year resident**	3 (11.1%)
**2nd year resident**	3 (11.1%)
**3rd year resident**	7 (25.9%)
**4th year resident**	9 (33.3%)
**5th year resident**	2 (7.4%)
**6th year resident**	2 (7.4%)
**Fellow**	1 (3.7%)
**Frequency of interventions assisted**	
**<1 case per month**	4 (14.8%)
**At least 1 case per month**	0 (0%)
**One case every 2 weeks**	6 (22.2%)
**One case every week**	9 (33.3%)
**>1 case a week**	8 (29.6%)
**Total # of cases assisted**	
**<10**	4 (14.8%)
**10–20**	6 (22.2%)
**20–50**	13 (48.1%)
**50–100**	2 (7.4%)
**100–200**	2 (7.4%)
**200–300**	0 (0%)
**>300**	0 (0%)

Supplementary Table S2 Differences in texture evaluation according to the surgeon's experience

**Item**	**Mean evaluation (/10)**
**Texture**	
**<100 cases operated**	6.70
**≥100 cases operated**	8.67
